# Shoulder scaption is dependent on the behavior of the different partitions of the infraspinatus muscle

**DOI:** 10.1007/s00276-020-02674-6

**Published:** 2021-01-19

**Authors:** Kyosuke Hoshikawa, Takuma Yuri, Hugo Giambini, Yoshiro Kiyoshige

**Affiliations:** 1 Department of Physical Therapy, Yamagata Prefectural University of Health Sciences, Yamagata, Japan; 2 Graduate School of Health Sciences, Yamagata Prefectural University of Health Sciences, Yamagata, Japan; 3 Department of Biomedical Engineering, The University of Texas at San Antonio, San Antonio, TX USA

**Keywords:** Infraspinatus muscle, Partition, Rotator cuff, Function, Shear-wave elastography

## Abstract

**Purpose:**

The purpose of this study was to investigate if the three partitions (superior, middle, and inferior partitions) of the infraspinatus muscle previously described in anatomical studies will present different behavior during scapular plane abduction** (**scaption**)** as described using shear-wave elastography, especially during initial range of motion.

**Methods:**

Eight volunteers held their arm against gravity 15° intervals from 30° to 150° in scaption. Shear-wave elastography was implemented at each position to measure shear modulus at rest and during muscle contraction, as a surrogate for muscle stiffness, of each partition. Muscle activity was defined as the difference in stiffness values between the resting positions and those during muscle contraction (Δ*E* = stiffness at contraction—stiffness at rest).

**Results:**

The activity value for the middle partition was 25.1 ± 10.8 kPa at 30° and increased up to 105° (52.2 ± 10.8 kPa), with a subsequent decrease at larger angle positions (*p* < .001). The superior partition showed a flatter and constant behavior with smaller activity values except at higher angles (*p* < .001). Peak activity values for the superior partition were observed at 135° (23.0 ± 12.0 kPa). Increase activity for inferior partition began at 60° and showed a peak at 135° (*p* < .001; 32.9 ± 13.8 kPa).

**Conclusion:**

Stiffness measured using shear-wave elastography in each partition of the infraspinatus muscle demonstrated different behavior between these partitions during scaption. The middle partition generated force throughout scaption, while the superior and inferior partitions exerted force at end range.

## Introduction

In the classic literature, the infraspinatus (ISP) muscle, one of the rotator cuff muscles, is defined as a thick triangular muscle occupying the ISP fossa [9]. The main function of the ISP muscle is to externally rotate the humerus [20] and generate a joint compression force in the gleno-humeral joint [30]. However, some investigators, using electromyogram (EMG), found that the ISP muscle acted as an abductor [19, 28]. In shoulder abduction motion, the rotator cuff muscles compress the humeral head to the glenoid against the superior migration action of the deltoid muscle. The activation timing of the supraspinatus muscle has been reported to be at 45° of abduction while that of the deltoid muscle at 60° [19]. If the ISP muscle effectively contributed to shoulder abduction, the ISP muscle should exert enough activation at initial range, prior to that of the deltoid muscle. Recent detailed anatomical studies demonstrated that the ISP muscle was anatomically divided into two or three distinct neuromuscular partitions based on differences in their attachment sites, muscle fiber directions, and intramuscular innervations [3, 6, 15, 22, 31]. Several authors demonstrated that the anterior-most region of the humeral insertion of the ISP muscle almost reached the anterior margin of the highest impression of the greater tuberosity [22–25, 31]. In addition, Kato et al. found that one of the partitions of the ISP muscle was innervated by branches arising from the supraspinatus muscle [15]. These findings from anatomical studies suggest that the partitions of the ISP muscle may independently play an important role in shoulder abduction.

While some investigators tried to measure independent activations of the partitions of the ISP muscle using EMG, it is difficult to measure the activation of each partition, especially in the inferior partition, throughout various shoulder motions as the fine wires can be displaced out of the muscle during contraction [1, 2, 4, 14]. Shear-wave ultrasound elastography (SWE) has been previously implemented to measure shear modulus of muscles, as a surrogate for muscle stiffness. Sasaki et al. reported a linear relationship between muscle shear modulus and tetanic muscular force produced by electrical stimulation in vivo [27]. Others have implemented SWE to measure the shear modulus of the supraspinatus muscle partitions and demonstrated differences in shear modulus among the various partitions [8, 10, 11]. These studies suggest SWE as a non-invasive imaging technique that can measure local stiffness of the rotator cuff muscles. Kuwahara et al., using elastography, investigated the functional differences in the partitions (superior, middle, and inferior partitions) of the ISP muscle during external rotation with 70° abduction [20]. They suggested that the superior partition contributes to abduction motion, the middle partition act as a prime external rotator, and the inferior partition has both functions. We hypothesized that the three partitions (superior, middle, and inferior partitions) of the ISP muscle previously described in anatomical studies [3, 6, 31] will present different behavior during scaption as described using SWE, especially during initial range of motion. A better understanding of the behavior of the individual infraspinatus muscle partitions during scaption using ultrasound elastography could provide beneficial information on patients who can hardly elevate their arms. The purpose of this study was to investigate if the three partitions (superior, middle, and inferior partitions) of the infraspinatus muscle previously described in anatomical studies would present different behavior during scaption as described using SWE.

## Methods

### Participants

A power analysis was performed a priori to calculate the sample size needed for one-way analysis of variance with repeated measures [effect size = 0.4, α error = 0.05, power = 0.95] using G* power 3.1 software (Heinrich Heine University, Duesseldorf, Germany) [7]. Thus, eight healthy male volunteers without any restrictions in their shoulder joints were recruited to this study after approval by our Institutional Ethics Review Board (#1609–15). Their mean age, body weight, and height were 20 ± 1 years old, 62 ± 2 kg and 174 ± 6 cm, respectively. The subjects’ experiences in sport were at a recreation level. Written informed consent was obtained from all participants.

### Muscle properties measurements

**Fig. 1:**
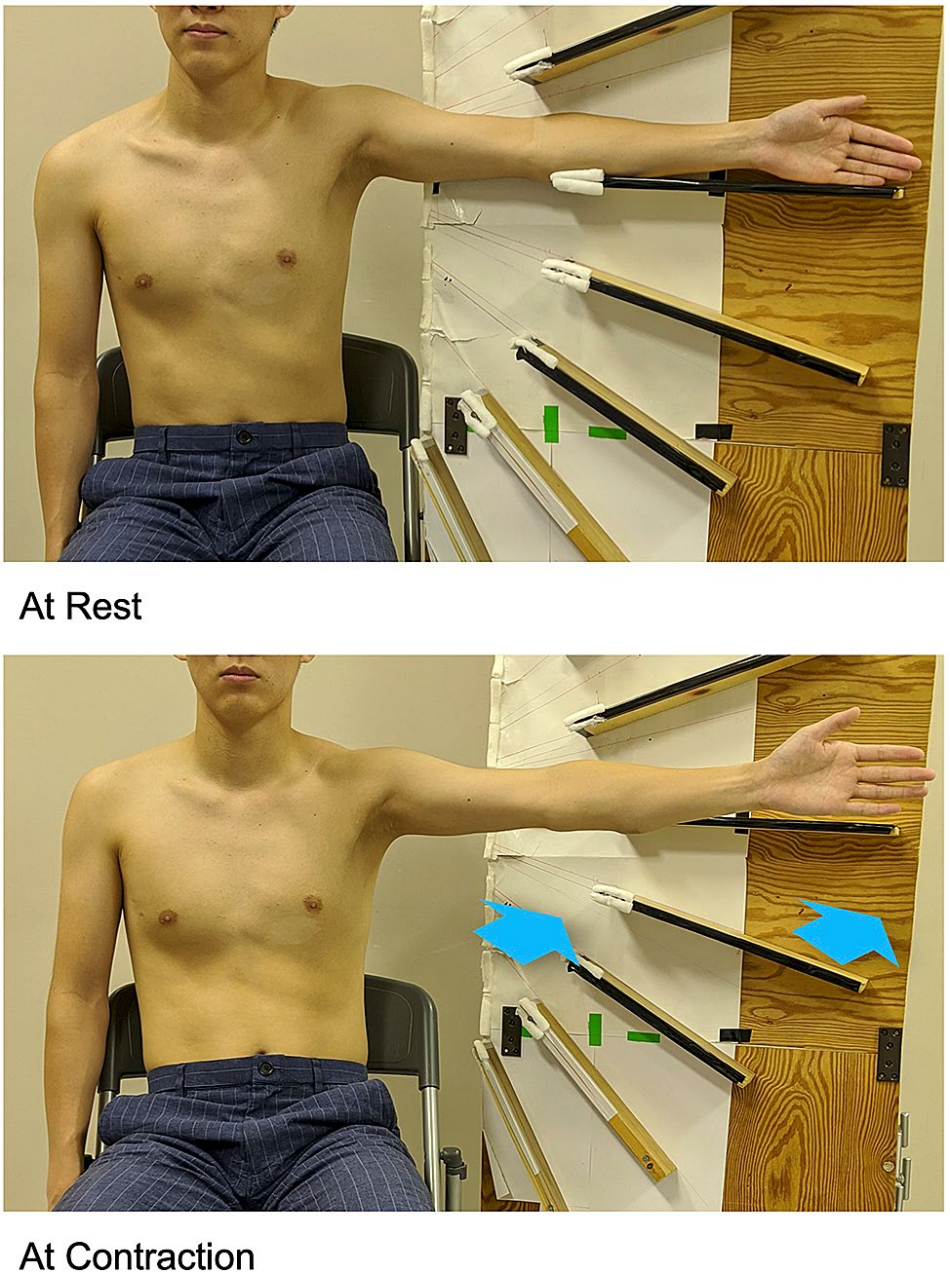
Scaption (abduction in scapular plane). SWE measurements of the ISP muscle were obtained 15° intervals from 30° to 150° using a hand-made semicircular protractor with shelves 15° intervals in scaption. At each position, the shear modulus at rest was measured while the participants place their arm on the shelves. The shear modulus at contraction was measured, while the tester moved the hand-made semicircular protractor back from the measurements at rest and participants hold their arms just against gravity at that position for approximately ten seconds

**Fig. 2:**
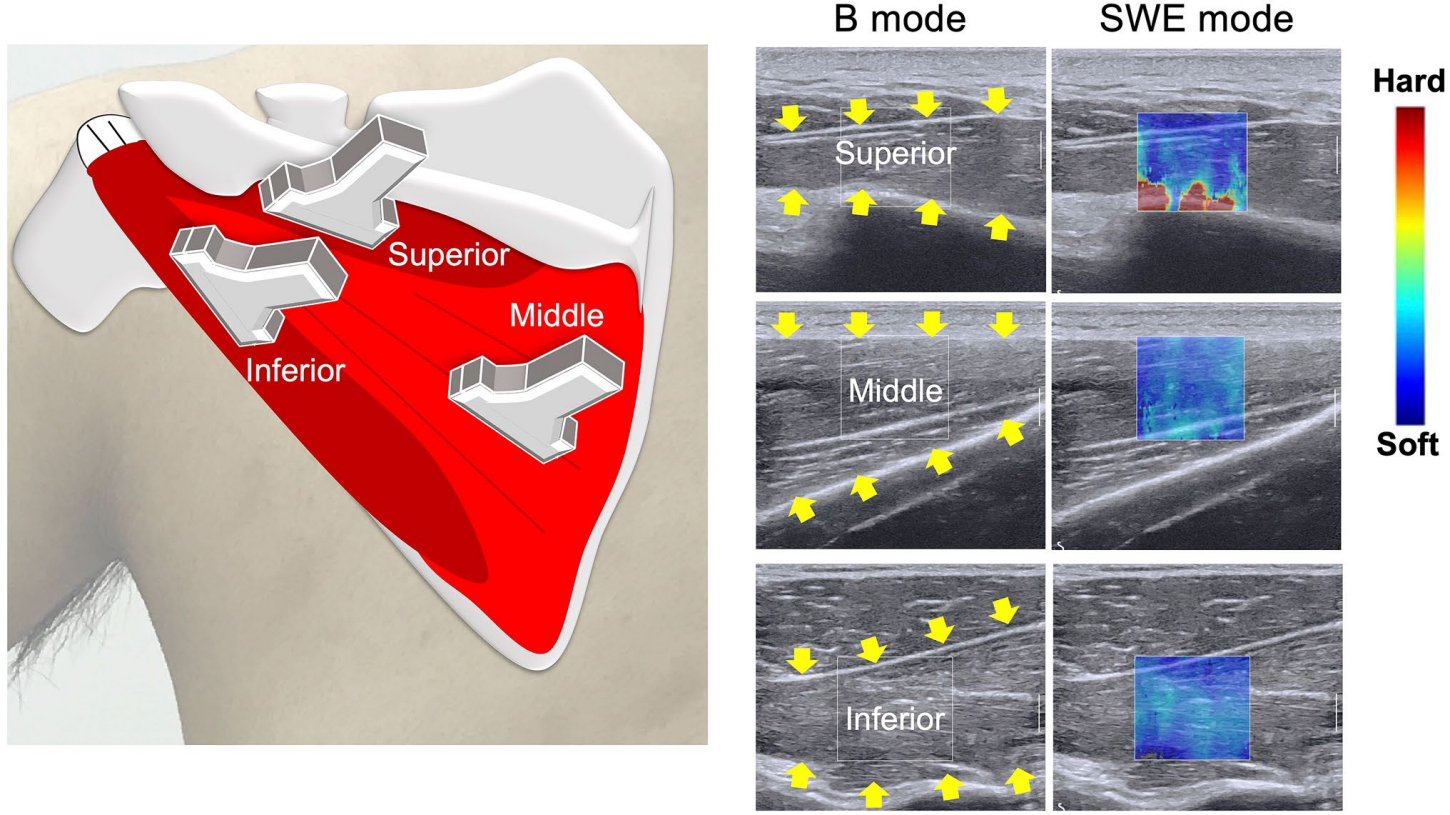
Probe orientations for the superior, middle, and inferior partitions of the infraspinatus muscle. Yellow arrows indicated the border of each partition. The SWE images were displayed in color-coded map with harder tissue represented in red and softer tissue in blue

An Aixplorer ultrasound scanner (Supersonic Imagine, Aix-en-Provence, France) and a 15–4 MHz linear array probe (SL 15-4, Vermon, France) were used to measure muscle shear modulus (kPa), as a surrogate for stiffness, in SWE mode (shoulder preset). The stiffness of the three partitions of the ISP muscle was obtained in the non-dominant arm of normal subjects. The subjects were instructed to sit on a chair and asked to rest their arms on a hand-made semicircular protractor with shelves 15° intervals from 30° to 150° in scapular plane abduction** (**scaption**)**. At each position, the stiffness at rest was measured while the participants place their arm on the shelves. The stiffness at contraction was measured while the tester moved the hand-made semicircular protractor back from the resting positions and participants held their arms against gravity at the same position for approximately ten seconds (Fig. 1). The ultrasound probe was gently placed on the skin atop each partition (superior, middle and inferior) of the ISP muscle (Fig. 2). Briefly, to obtain the B-mode image for the superior partition, the probe was placed 1 cm down and parallel to the scapula spine. For the middle partition, the probe was placed mid-point of the medial border of the scapula. For the inferior partition, at first, the fascia between the inferior partition of the ISP and teres minor muscles was visualized. Then, the probe was placed on the region of teres minor muscles [3, 6, 20, 31]. SWE images were simultaneously displayed on the B-mode image as a square color image. To measure the stiffness, three random images were chosen from the continuous video recording during the measurements. Three regions of interest were placed at the center of each specific muscle partition to obtain stiffness outcomes based on the shear wave speed [29]. This process was repeated for each shoulder position. The measurements were conducted by a single sonographer (KH). Muscle activity was defined as the difference in stiffness values between the resting positions and those during muscle contraction (Δ*E* = stiffness at contraction − stiffness at rest) [20, 32, 33].

### Statistical analysis

SPSS statistical software (version 24.0; SPSS, Chicago, IL, USA) was used for all the statistical analyses. Intraclass correlation coefficient (ICC_1,3_) was implemented to evaluate the intra-rater reliability of three images between eight subjects based on the ICC form developed by Koo and LI (one-way random effects, absolute agreement, multiple measurements) [18]. Reliability was classified as poor (less than 0.50), moderate (between 0.50 and 0.75), good (between 0.75 and 0.90), and excellent (greater than 0.90). Because the Shapiro–Wilk test did not indicate a normal distribution of the data, non-parametric tests were conducted. Friedman with Dunn's post hoc tests was used to evaluate differences in measurement outcomes among angles during scaption in three partitions. Statistical significance was set to *p* < 0.05.

## Results

**Fig. 3:**
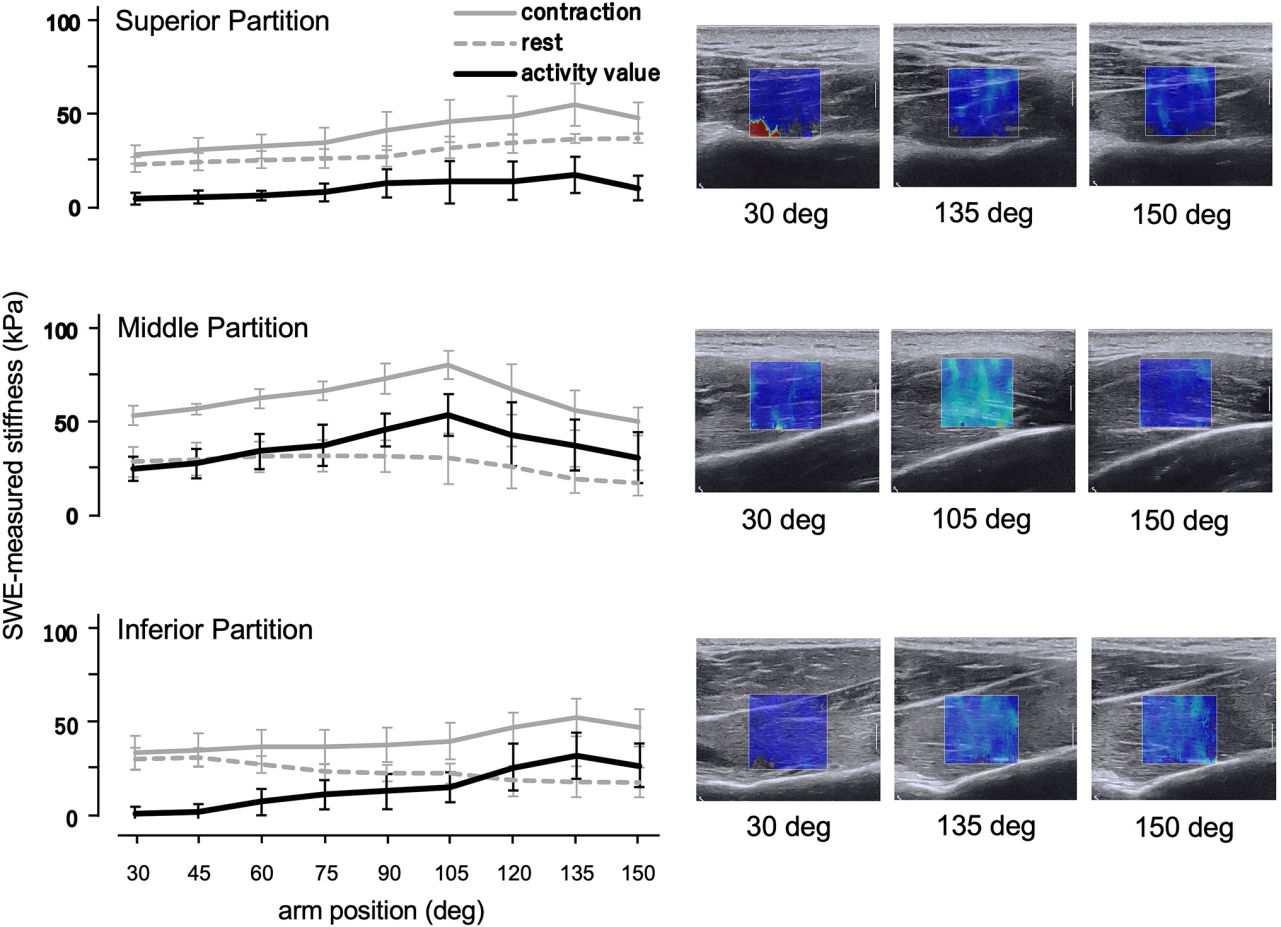
The shear modulus at rest, at contraction and activity value for each partition during scaption at various shoulder positions. The representative SWE images were chosen at 30°, peak position (135° for superior and inferior partitions, 105° for middle partition), and 150° from measurements for shear modulus at contraction

ICC_1,3_ analysis resulted in excellent reliability (0.994–0.999). Stiffness values at rest (mean ± SD) in both middle and inferior partitions resulted in peak outcomes at 45° (32.5 ± 7.5 kPa for middle partition and 31.4 ± 7.7 kPa for inferior partition), decreasing with increasing scaption angles (*p* = 0.005 for middle partition and *p* = 0.010 for inferior partition). Stiffness of the superior partition showed a constant increase in values with shoulder scaption positions (*p* < 0.001; Fig. 3). Peak stiffness at rest of the superior partition was 32.6 ± 9.1 kPa at 150°.

Stiffness for the middle partition during muscle contraction increased up to 105° (81.9 ± 10.7 kPa), with a subsequent decrease (*p* < 0.001). The superior and inferior partitions showed a flatter and constant behavior compared to the middle partition, except at higher shoulder angles. Peak stiffness of the superior and inferior partitions were 55.0 ± 10.5 kPa at 135°, and 52.4 ± 12.9 kPa at 135°, respectively (*p* < 0.001 for both superior and inferior partitions; Fig. 3).

The activity value for the middle partition was 25.1 ± 10.8 kPa at 30° and increased up to 105° (52.2 ± 10.8 kPa), with a subsequent decrease at larger angle positions (*p* < 0.001). The superior partition showed a flatter and constant behavior with smaller activity values except at higher angles (*p* < 0.001). Peak activity values for the superior partition was observed at 135° (23.0 ± 12.0 kPa). Increase activity for inferior partition began at 60° and showed a peak at 135° (*p* < 0.001; Fig. 3; 32.9 ± 13.8 kPa).

## Discussion

The purpose of the current study was to investigate the behavior of the superior, middle, and inferior partitions of the ISP muscle during scaption at rest and during muscle contraction in vivo using SWE. As we hypothesized, the shear modulus, as a surrogate for stiffness, measured using SWE in each partition of the ISP muscle demonstrated different behavior during scaption. To our knowledge, this is the first study to investigate the functional behaviors in three anatomical partitions of the ISP muscle during scaption.

We obtained three quantitative outcomes in the three partitions of the ISP muscle; stiffness at rest, during muscle contraction, and the activity value representing the difference between these two. As muscle is stretched, it will tend to increase its passive resistance to deformation. Koo et al. showed a linear relationship between shear modulus measured by SWE and passive force [17]. Thus, the stiffness at rest can indicate the length of muscle. Stiffness at rest from the superior partition gradually increased in this study. This indicates that the superior partition was gradually elongated with increasing elevation angles. On the other hand, stiffness at rest for the middle and inferior partitions gradually decreased, indicating that these partitions shortened from the starting and elongated position. SWE-measured stiffness during muscle contraction is the sum of the stiffness at rest and those measured during voluntary contraction. Therefore, to estimate the stiffness generated by voluntary contraction, we defined the difference in stiffness at rest and during contraction (Δ*E* = stiffness during contraction − stiffness at rest) as activity value. When the measured stiffness at rest results in substantial values due to its physical elongated condition, this will in turn yield low activity outcomes, with the muscle acting as a dynamic tenodesis. This indicates that the inferior partition acted as dynamic tenodesis during the initial range of motion. The activity value of inferior partition gradually increased from 60° up to 135°. The superior partition became tight with increasing elevating angles and had small activity value with a peak outcome at 135°. On the contrary, the middle partition was the only partition of the three not showing low activity values from the initial range to end range, with a peak at 105°.

EMG studies have demonstrated that ISP muscle activation is described by a trapezoidal behavior with a peak value at late mid-range of scaption in a no-load type condition [12, 21]. These results coincide with the activity values in the middle partition observed in our study. Some investigators measured independent activation of those partitions using EMG, and concluded that the muscle partitions have different roles [1, 2, 4, 14]. However, EMG measurements from the partitions were not obtained during scaption, rather during specific positions, e.g. empty can test. On the other hand, Alenabi et al. 2019 [2] measured EMG in the superior and middle partitions of the ISP muscle at 30°, 90°, and 150° during scaption and demonstrated that the activation of both the superior and middle partitions of the ISP muscle, with 50% maximal voluntarily contraction, increased with increasing angle. These results are not in line with those obtained in the current study for the middle partition. One possible explanation is the difference in the strength measurements. The authors applied 50% maximal voluntary contraction, while in the current study we only considered the force induced by having the subject hold the arm against gravity.

Previous kinematic studies have reported that the rotator cuff muscles exert gleno-humeral joint compression force during scaption to maintain the humeral head in the glenoid fossa, especially at initial range of scaption against the superior migration force produced by the deltoid muscle. Yanagawa et al. demonstrated that the ISP muscle exerts compression force throughout scaption [30]. The activity value behavior of the middle partition was similar to Yanagawa’s outcomes. However, the activity values of the superior and inferior partitions were small during the initial range and peak values were observed at 135°. Hawkes et al. showed that the deltoid muscle induces an inferior shear force at the end range of scaption, and that this force is balanced by the rotator cuff muscles [12]. Thus, the superior and inferior partitions of the ISP may exert this type of balancing force suggested by Hawkes et al. at the end range of scaption.

Several authors reported that the anterior-most region of the humeral insertion of the ISP muscle almost reached the anterior margin of the highest impression of the greater tuberosity [22–25, 31]. Kato et al. demonstrated that the ISP muscle was comprised of two parts, namely the transverse and oblique parts [15]. They found that the transverse part only has a tendinous membrane which is attached to the tendinous portion of the oblique part [15]. Based on these findings, they suggested that the oblique part mainly contributes to shoulder abduction because the tendinous portion of its superior region reaches the anterior-most area of the greater tuberosity, and that the transverse part may provide support and stabilize the tendinous portion of the oblique part during shoulder motion [15]. The difference in the tendinous insertion between the transverse and oblique parts may explain the differences in at-rest values of three partitions, namely, the activity value of the superior partition increased with increasing elevation angle while those of the middle and inferior partitions decreased. Reed et al., using EMG, demonstrated the supraspinatus and ISP muscles had similar initial activation timing during scaption [26], and Yanagawa et al. showed less muscle force production in the supraspinatus muscle than the ISP muscle at initial range of scaption [30]. These previous results also support the function of middle partition as an initiator of scaption.

Clinically, a rotator cuff tear is one of the most common musculoskeletal disorders. Studies report the tear to include the supraspinatus tendon and/or junction of the supraspinatus and ISP tendons [13, 16]. Interestingly, some patients with a rotator cuff tear can elevate their arms, even though the supraspinatus tendon is completely torn, while some patients whose tear extended to the ISP tendon can hardly elevate their arm. One possible explanation for this is the compensatory effect imposed by the ISP muscle. If the ISP muscle can exert a joint compression force large enough to suppress the superior migration force produced by the deltoid muscle at the initial range of scaption, the arm could be elevated. Yuri et al. found that the middle partition has a firm capsular attachment area, while the superior and inferior partitions had less or no firm capsular attachment areas and bony insertions to the greater tuberosity [31]. Clark et al. suggested that the firm capsular attachment area could contribute to a distribution of the tension to the humeral head [5]. The results of our study indicated that the middle partition might act as the initiator of scaption via the bony insertion of the greater tuberosity and exert enough joint compression force via its capsular attachment.

While EMG and kinematic evaluations have been adopted for assessing muscular function, the former is invasive and has not been successfully implemented in the inferior partition due to wire displacement [1], and the latter has not been applied in the various muscular partitions. In our study, we successfully discriminated the spatio-temporal relationship of the three partitions of the ISP muscle during scaption using SWE. We showed that the middle partition may play a substantial role in initiation of scaption, the inferior partition could act in a dynamic tenodesis manner, and the superior and inferior partitions may oppose the function of the deltoid muscle at the end range of scaption. Additionally, estimates of muscular elongation and shortening can be obtained by quantifying stiffness at rest. These outcomes suggest SWE as a novel technique for precise assessment of the properties of the individual muscular partitions.

There are some limitations in this study. First, the subjects included only young males. Therefore, further studies are needed to extrapolate our findings in clinical setting to an older population presenting different pathologies. Second, the strength at contraction in this study corresponded to the manual muscle test grade 3, namely, subjects held their arms against gravity. While this process could be considered as a weak muscle contraction, it allowed for the evaluation of the independent activations of the ISP muscle partitions. Future studies should evaluate other shoulder positions and loading conditions, as well as their effect on ISP muscle behavior to verify the functional role of different partitions of the ISP muscle.

## Conclusion

Stiffness measured using shear-wave elastography in each partition of the ISP muscle demonstrated different behavior between these partitions during scaption. The middle partition generated force throughout scaption, while the superior and inferior partitions exerted force at end range.
